# Effect of ambient fine particulates (PM_2.5_) on hospital admissions for respiratory and cardiovascular diseases in Wuhan, China

**DOI:** 10.1186/s12931-021-01731-x

**Published:** 2021-04-28

**Authors:** Zhan Ren, Xingyuan Liu, Tianyu Liu, Dieyi Chen, Kuizhuang Jiao, Xiaodie Wang, Jingdong Suo, Haomin Yang, Jingling Liao, Lu Ma

**Affiliations:** 1 Wuhan University School of Health Sciences, No. 115 Donghu Road, Wuchang district, Wuhan, 430071 Hubei China; 2 Wuhan Information Center of Health and Family Planning, Wuhan, 430021 China; 3 Department of Biostatistics, Yale University, New Haven, CT 06520 USA; 4 Department of Nutrition and Food Hygiene, School of Public Health, Medical College, Wuhan University of Science and Technology, No. 2 Huangjiahu West Road, Hongshan district, Wuhan, 430081 Hubei China

**Keywords:** Air pollution, Particulate matter, Spatial epidemiology, Case-crossover study

## Abstract

**Background:**

Positive associations between ambient PM_2.5_ and cardiorespiratory disease have been well demonstrated during the past decade. However, few studies have examined the adverse effects of PM_2.5_ based on an entire population of a megalopolis. In addition, most studies in China have used averaged data, which results in variations between monitoring and personal exposure values, creating an inherent and unavoidable type of measurement error.

**Methods:**

This study was conducted in Wuhan, a megacity in central China with about 10.9 million people. Daily hospital admission records, from October 2016 to December 2018, were obtained from the Wuhan Information center of Health and Family Planning, which administrates all hospitals in Wuhan. Daily air pollution concentrations and weather variables in Wuhan during the study period were collected. We developed a land use regression model (LUR) to assess individual PM_2.5_ exposure. Time-stratified case-crossover design and conditional logistic regression models were adopted to estimate cardiorespiratory hospitalization risks associated with short-term exposure to PM_2.5_. We also conducted stratification analyses by age, sex, and season.

**Results:**

A total of 2,806,115 hospital admissions records were collected during the study period, from which we identified 332,090 cardiovascular disease admissions and 159,365 respiratory disease admissions. Short-term exposure to PM_2.5_ was associated with an increased risk of a cardiorespiratory hospital admission. A 10 μg/m^3^ increase in PM_2.5_ (lag0–2 days) was associated with an increase in hospital admissions of 1.23% (95% CI 1.01–1.45%) and 1.95% (95% CI 1.63–2.27%) for cardiovascular and respiratory diseases, respectively. The elderly were at higher PM-induced risk. The associations appeared to be more evident in the cold season than in the warm season.

**Conclusions:**

This study contributes evidence of short-term effects of PM_2.5_ on cardiorespiratory hospital admissions, which may be helpful for air pollution control and disease prevention in Wuhan.

**Supplementary Information:**

The online version contains supplementary material available at 10.1186/s12931-021-01731-x.

## Background

Air pollution has remained an important global health issue [1]. Numerous epidemiological studies have proven that PM_2.5_, particulate matter with an aerodynamic diameter less than 2.5 μm, is a critical contributor that leads to increased mortality and morbidity [2, 3]. According to the analysis of the Global Burden of Diseases Study, approximately 2.94 million deaths and 10.5 million disability-adjusted life years (DALYs) globally are attributable to ambient particulate matter pollution, making it the eighth leading risk for death [4].

Previous studies have provided strong evidence of the harmful effects of PM_2.5_ on cardiorespiratory diseases [5, 6]. Although several large-scale studies, conducted in western developed countries [7, 8], have examined the associations between air pollution and cardiorespiratory hospital admissions, these results may not be applicable to developing countries due to local climate conditions, PM chemical components and population susceptibility. In China, several large-scale analyses have been conducted [9, 10]. However, the populations of these studies were obtained by specific sampling methods and may not represent the entire population. Some single-center epidemiological studies have been conducted in several large cities in China [11, 12], but the hospital admissions data of these studies were collected from a limited number of hospitals, which may introduce selection bias. Therefore, studies that examine the association between PM_2.5_ and cardiorespiratory hospital admissions based on all citizens of a large city are needed to better understand the real impact of ambient fine particulate matter in China.

Exposure assessment methods are crucial for epidemiological studies. Commonly used air pollution assessment methods include monitoring data derived from fixed stations, Dispersion Models (DM), atmospheric Chemical Transport Models (CTMs), and Land Use Regression (LUR) [13, 14]. Most studies have estimated individual exposure to air pollution using the ambient concentrations derived from fixed stations, which lacks spatial and temporal resolution. Conventional DM and CTMs require various data with high precision, which makes the simulation process complicated and high-cost [15]. Compared with the above methods, LUR models, which use land use, geographic, and traffic characteristics to explain spatial variations of air pollution concentrations, have proven to be cost-effective methods of air pollution exposure assessment. With the development of geographic information system (GIS) technology, LUR has achieved great success, mainly in Europe and North America [16, 17]. In China, however, only a few studies have applied LUR models in epidemiological research.

Therefore, this study was conducted based on the admission data of all hospitals in Wuhan, from October 2016 to December 2018. Considering Wuhan’s universal access to hospital healthcare and the availability of these records, the impact of PM_2.5_ on the entire population can be assessed. Furthermore, LUR models were developed to better capture individual PM_2.5_ exposure. The objective of this analysis was to examine the association between PM_2.5_ and cardiorespiratory hospital admissions.

## Methods

### Study area

With a land area of 8569.15 km^2^ and a population of about 10.9 million (Wuhan Statistical Yearbook-2018), Wuhan (29.58°N to 31.22°N and 113.41°E to 115.05°E) is the capital city of Hubei Province and a megacity in central China. Due to its subtropical, monsoon climate, Wuhan has typical weather featured in distinct seasons and abundant rainfall. The major sources of air pollution in the city are biomass and coal combustion, steel manufacture, smelting, and vehicle emissions [18].

### Case ascertainment

Daily hospitalization records were obtained from the Wuhan Information center of Health and Family Planning (http://wjw.wuhan.gov.cn/) between Oct 1, 2016 and Dec 31, 2018. The Wuhan Information center of Health and Family Planning is a hospital authority within the municipal Bureau of Health, to which all the hospital**s** in Wuhan have to report their information of hospital infrastructure, medical service and management. All of the public hospitals (university affiliated hospitals, regional hospitals, provincial hospitals and so on), a total of 59 municipal hospitals, were included in this study. From each record, we extracted de-identified patient age, sex, home address, and primary diagnosis. The diagnoses were made by licensed specialized physicians according to current clinical guidelines. Cardiorespiratory hospital admissions in the present study were identified based on the primary diagnosis according to the International Classification of Diseases, 10th Revision (ICD-10): total cardiovascular disease (CVD, I00–I99), hypertension (I10–I15), coronary heart disease (CHD, I20–I25), stroke (I60–I69), total respiratory disease (J00–J99), and chronic obstructive pulmonary disease (COPD, J41–J44). A total of 2,806,115 hospital admission records were collected during the study period, from which we identified 332,090 for total cardiovascular diseases and 159,365 for total respiratory diseases. Specific inclusion and exclusion criteria are outlined in Additional file 1: Figure S1. The present study is considered exempt from institutional review board approval since the data used were collected for administrative purposes without any personal identifiers.

### Air pollutant data

**Fig. 1:**
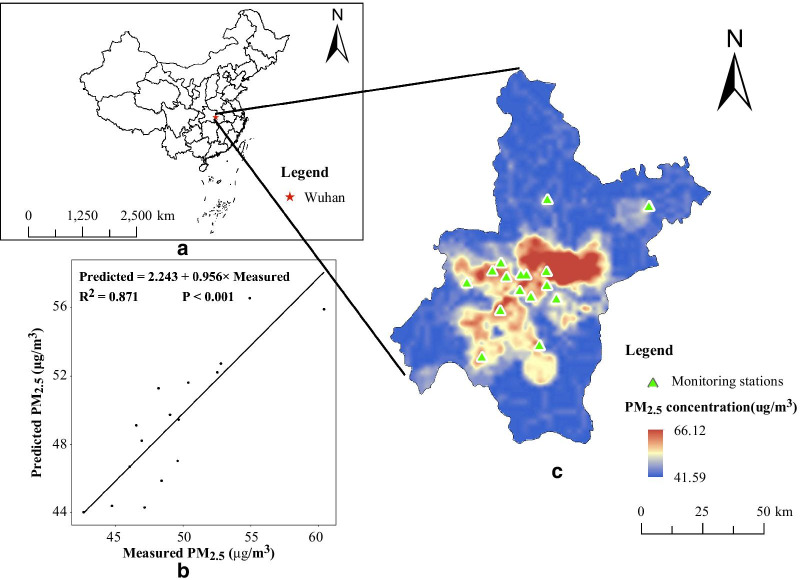
Study area and the results of LUR models. **a** Geographical location of Wuhan in China. **b** Spatial distribution of mean PM_2.5_ estimations across Wuhan city from October 1, 2016 to December 31, 2018. **c** Scatter plot correlating the measured and predicted PM_2.5_ values from 16 monitoring stations

During the study period, the air pollution data were collected from the Wuhan Environmental Protection Bureau (http://hbj.wuhan.gov.cn/), which has established 20 ambient air quality monitoring stations in the 13 districts of Wuhan city. To calculate daily 24-h concentrations, ≥ 75% of the 1-h values must have been available on that particular day; To calculate the annual concentration, there must be at least 324 daily values available. Four stations were excluded because the above criteria were not met. Finally, daily 24-h average concentration data for PM_2.5_ (unit, μg/m^3^), sulfur dioxide (SO_2_) (unit, μg/m^3^), nitrogen dioxide (NO_2_) (unit, μg/m^3^), and carbon monoxide (CO) (unit, mg/m^3^) during the study period were collected from 16 air quality monitoring stations (Fig. 1). Daily meteorological data including mean temperature (°C) and relative humidity (%) during the study period were collected from the China Meteorological Data Network (http://data.cma.cn/).

### LUR model

In this study, LUR models were constructed by combining measurements of PM_2.5_ from fixed-site monitors with a range of geographic predictors. The detailed model-building process is described in the supplementary materials (Additional file 2: Table S1).

Final models were represented with 1 km spatial resolution. Kriging interpolation was used to transform predicted PM_2.5_ data from monitors into concentration maps (Fig. 1). We then extrapolated annual-mean PM_2.5_ concentrations from the LUR model to daily levels, following the method described in previous studies [19]. Briefly, we geocoded individual addresses and assigned the annual average PM_2.5_ concentrations from the LUR models to each individual. Daily PM_2.5_ concentrations assigned to each subject were adjusted by the ratio of daily-specific PM_2.5_ concentrations to the estimated annual average PM_2.5_ concentrations at the nearest monitor.

### Statistical design

The case-crossover (CCO) design was first proposed by Maclure [20] to study transient effects on the risk of acute events. As each subject serves as his or her own control, this type of study controls for the influence of self-confounding variables that remain constant.

In this study, we performed a time-stratified case-crossover study design to evaluate associations between short-term PM_2.5_ exposures and cardiorespiratory hospital admissions in Wuhan. The case day was defined as the day of hospital admission and the control days were identified by matching the day of the week (DOW) within the same year and month. By virtue of this design, the potential confounding effects of long-term trends and seasonality can be largely eliminated.

### Analytic model

We used a conditional logistic regression (CLR) model to obtain estimates of the odds ratios (ORs) and 95% confidence intervals (CIs) for the effect of PM_2.5_ exposures on cardiorespiratory hospital admissions. To control covariates, we applied a natural cubic spline (NCS) function with 3 degrees of freedom (df) for both temperature and humidity to eliminate nonlinear confounding effects.

Considering that a single-day lag model might underestimate the association [21], the cumulative effects were estimated using different lag structures, including both single-day (lag0 to lag6) and several days’ moving averages (lag0–1 to lag0–6). Linearity for exposure–response relationship between PM_2.5_ and cardiorespiratory admissions was further checked by smoothing the PM_2.5_ terms using the NCS function (with 3 df).

Furthermore, we conducted stratification analyses by age (< 45, 45–54, 55–64, 65–74, and > 74 years), sex (male and female), and season (warm: April to September; cold: October to March of the next year) to explore the potential effect modifiers on the associations between PM_2.5_ and cause-specific hospital admissions deriving from the single pollutant model. The Z-test was applied to test the statistical significance of differences by gender or season [22].

### Sensitivity analysis

To check the robustness of our main results, we conducted several sensitivity analyses by: (1) fitting two-pollutant models by additionally adjusting for air pollutants (NO_2_, SO_2_, CO) collected from the monitoring stations closest to patients’ homes; (2) conducting a symmetric CCO design (days: ± 7, 14) [23]; and (3) changing the degrees of freedom of meteorological variables (2–4 df).

All of the analyses were conducted using R, version 3.5.1. We used the “survival” package for CLR analysis. Two-sided tests were conducted, and effects with p < 0.05 were considered to be statistically significant. All results of model estimates are reported as ORs and 95% confidence intervals (CIs) associated with each 10 μg/m^3^ increase in PM_2.5_ concentrations.

## Results

A total of 491,455 hospital admissions, of which 332,090 were for total cardiovascular diseases and 159,365 for total respiratory diseases, were recorded from October 1, 2016 to December 31, 2018 in Wuhan (Table 1). The mean age of cardiovascular disease admissions was 63.69 years (SD = 17.58) and that of respiratory diseases admissions was 68.17 years (SD = 10.42). For both cardiovascular and respiratory admissions, older people over 74 years old accounted for the largest proportion, and the number of males was higher than that of females during the study period.

**Table 1:** Basic characteristics of cardiovascular disease and respiratory disease admissions in Wuhan (Oct 1, 2016 to Dec 31, 2018)

Characteristic	Cardiovascular diseases (n = 332,090)	Respiratory diseases (n = 159,365)
Age [mean ± SD (years)]	63.69 ± 17.58	68.17 ± 10.42
Age group [n (%)]		
< 45	20,089 (6)	25,212 (16)
45–54	41,956 (13)	18,507 (12)
55–64	79,240 (24)	32,206 (20)
65–74	85,869 (26)	35,292 (22)
> 74	104,936 (31)	48,148 (30)
Sex [n (%)]		
Men	181,133 (55)	90,961 (57)
Women	150,957 (45)	68,404 (43)
Sub-diagnoses [n (%)]		
COPD		41,467 (26)
Hypertension	51,790 (16)	
CHD	97,846 (29)	
Stroke	113,967 (34)	
Season at admission [n (%)]		
Warm	163,495 (49)	74,358 (47)
Cold	168,595 (51)	85,007 (53)

*SD* standard deviation, *warm* April to September, *cold* October to March of the next year

**Fig. 2:**
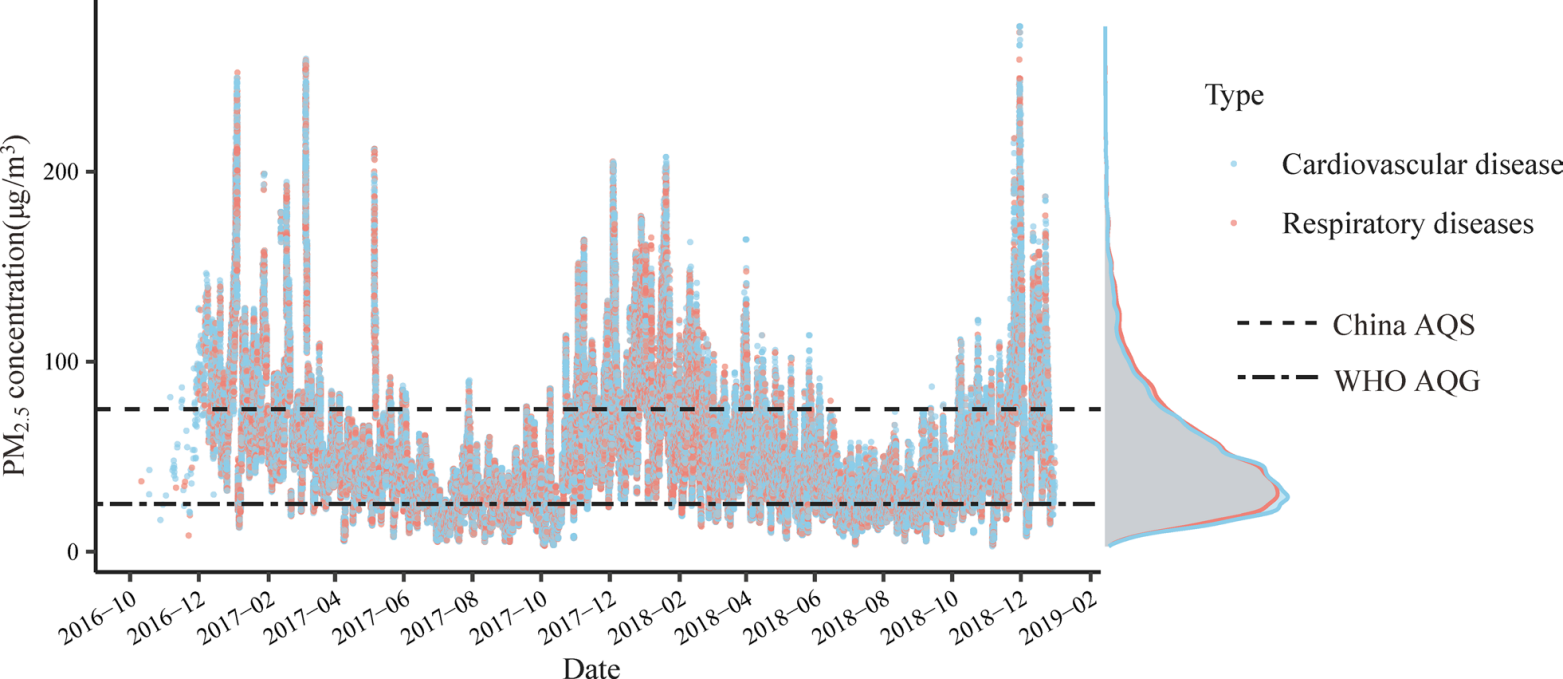
Distribution of individual PM_2.5_ exposures on the day of admission. Data points represent the PM_2.5_ concentrations of individuals. The density plot on the right margin of the y-axis visualizes the distribution of the number of subjects over the PM_2.5_ concentration. The dashed lines denote the Grade II criteria set by the Chinese AQS (75 μg/m^3^) and the WHO AQG (25 μg/m^3^)

During the study period, the mean daily average concentrations were 48.2 μg/m^3^ for PM_2.5_ and the mean daily average temperature and humidity were 16.3 °C and 80% respectively (Additional file 3: Table S2), reflecting the subtropical climate in Wuhan. Figure 2 shows seasonal trends of PM_2.5_ concentrations, with high values in winter and low values in summer. The PM_2.5_ exposure of most patients was within China’s ambient air quality standards (AQS), but it exceeded the World Health Organization (WHO) air quality guidelines (AQG).

**Fig. 3:**
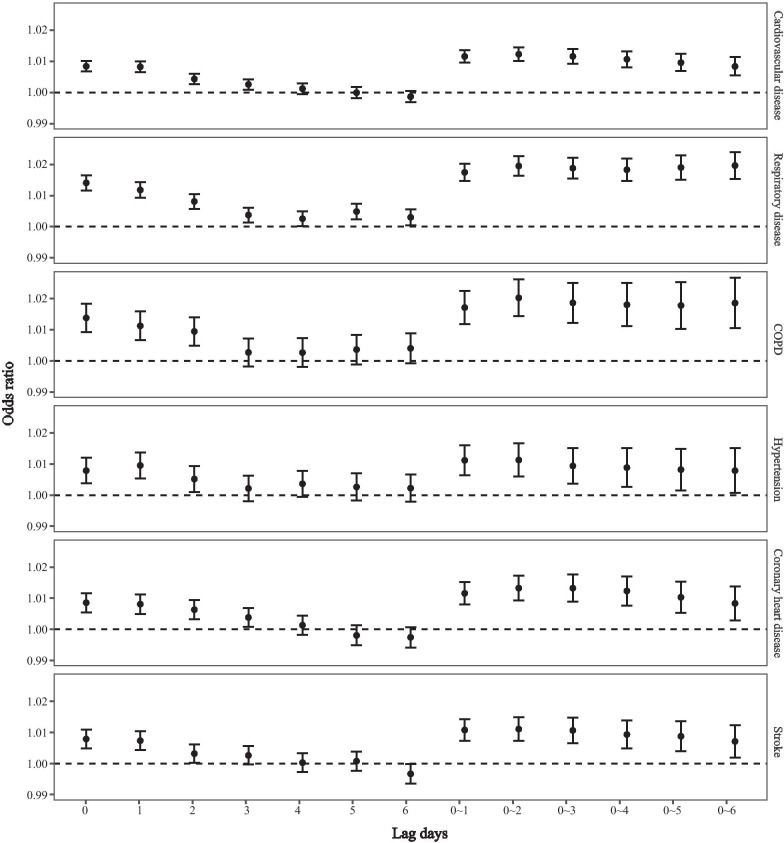
OR (95% CI) of hospital admission for total and cause-specific cardiorespiratory disease per 10 μg/m^3^ increase of PM_2.5_ with different lag patterns in single-pollutant models

PM_2.5_ showed similar lag patterns for it’s impact on total cardiorespiratory hospital admissions (Fig. 3). Detailed risk estimates are listed in the supplementary materials (Additional file 5: Table S4). For single-day lags, a weakened lagging effect of PM_2.5_ was observed from lag0 to lag6. Significant harmful effects were shown on lag0–lag2 with respect to the risk of admissions for all cardiorespiratory diseases, and the highest risks were found at lag0, except for hypertension. For the cumulative lag day effect, we found significant positive associations in all analyzed hospital admissions, while the greatest effects for all diseases were observed at lag0–2. Thus, in the subsequent analyses, we mainly chose lag0–2 as the exposure period to evaluate the acute effects of ambient particulate matter. The moving average lag model usually had higher estimates than that of single-day exposure. Each 10 μg/m^3^ increase in PM_2.5_ at lag0–2 was associated with a 1.2% (95% CI 1.0%–1.4%) increment in daily hospital admissions for total CVD and a 2.0% (95% CI 1.6%–2.3%) increment for total respiratory diseases (Additional file 5: Table S4). The effect estimates remained stable in the symmetric CCO design (Additional file 6: Table S5) and with different degrees of freedom for smoothing of meteorological variables (2–4 df) (Additional file 7: Table S6).

**Fig. 4:**
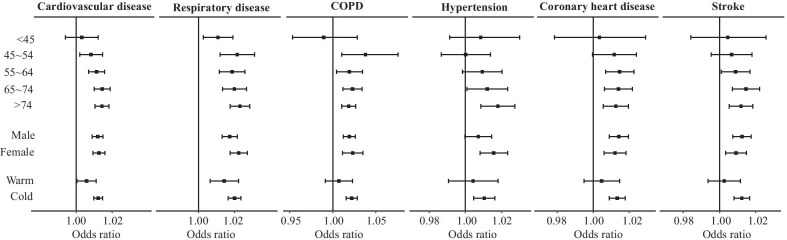
OR (95% CI) of hospital admission per 10 μg/m^3^ increase of PM_2.5_ stratified by age, gender, and season at admission at lag0–2

For subgroup analysis, we examined the associations between PM_2.5_ and cardiorespiratory hospital admissions at lag0–2, classified by age, gender, and admission season (Fig. 4). In age-specific analyses, positive associations were found in all age groups for respiratory admission. Stronger effects of PM_2.5_ on both cardiovascular and respiratory admission were observed in the elderly (over 65 years). However, the hazard effects among people aged > 74 years were slightly lower than that of people aged 65–74 years in some cause-specific diseases (COPD, coronary heart disease, and stroke). In addition, COPD patients aged 45–54 years were at the greatest risk, with ORs of 1.042 (95% CI 1.010–1.075) (Additional file 8: Table S7). In sex-specific analyses, exposures to PM_2.5_ showed significant effects on both genders, except hypertension, but gender differences in PM-associated risks were statistically insignificant. In season-specific analyses, we found a greater effect of PM_2.5_ for all cardiorespiratory diseases in the cold season than in the warm season.

**Fig. 5:**
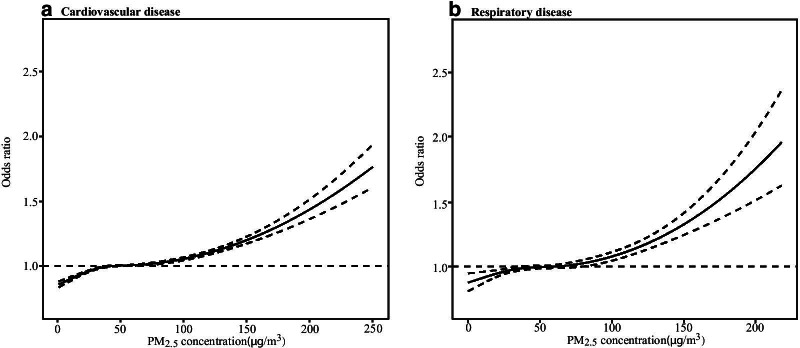
Concentration response relationship of PM_2.5_ concentrations with hospital admissions for cardiorespiratory diseases at lag0–2. The solid line represents the predicted OR, and the dotted lines represent the 95% CI

There was a clear dose–response relationship between PM_2.5_ concentration and hospital admissions for both cardiovascular and respiratory diseases (Fig. 5). Both results exhibited generally similar patterns. The relationship was approximately linear, with a tiny fluctuation at lower concentrations (< 100 μg/m^3^) and a sharper response at higher concentrations.

The risk estimates of PM_2.5_ with a three-day moving average (lag0–2) in two-pollutant models were summarized in Table 2, adjusting for other air pollutants (NO_2_, SO_2_ and CO). Overall, the effects of PM_2.5_ remained stable after adjusting for gaseous pollutants for total cardiorespiratory diseases, while the effect estimates of PM_2.5_ in two-pollutant model were slightly smaller than in single-pollutant model. Notably, for three gaseous pollutants, the adverse effects were observed on most cardiorespiratory diseases after adjusting for PM_2.5_.

**Table 2:** Odds ratio (95% CIs) of admissions from total CVD, respiratory, COPD, hypertension and stroke per 10 μg/m^3^ increase in a 3-day moving average (lag 0–2) concentration of PM_2.5_, with and without adjustment for pollutants

Pollutants	CVD	Respiratory	COPD	Hypertension	CHD	Stroke
PM_2.5_^a^	1.012 (1.010, 1.014)	1.020 (1.016, 1.023)	1.020 (1.014, 1.026)	1.011 (1.006, 1.017)	1.013 (1.009, 1.017)	1.011 (1.007, 1.015)
PM_2.5_ + NO_2_^b^						
PM_2.5_	1.003 (1.001, 1.005)	1.011 (1.007, 1.015)	1.012 (1.005, 1.019)	1.001 (0.994, 1.007)	0.999 (0.995, 1.005)	1.006 (1.001, 1.010)
NO_2_	1.002 (1.001,1.002)	1.002 (1.001, 1.002)	1.002 (1.001, 1.003)	1.002 (1.002, 1.003)	1.003 (1.002, 1.003)	1.001 (1.001, 1.002)
PM_2.5_ + SO_2_^b^						
PM_2.5_	1.009 (1.007, 1.012)	1.013 (1.010, 1.017)	1.012 (1.005, 1.018)	1.009 (1.003, 1.015)	1.009 (1.004, 1.013)	1.011 (1.007, 1.015)
SO_2_	1.001 (1.001, 1.002)	1.003 (1.002, 1.003)	1.004 (1.003, 1.005)	1.001 (0.999, 1.002)	1.002 (1.001, 1.003)	1.001 (0.999, 1.001)
PM_2.5_ + CO^b^						
PM_2.5_	1.008 (1.006, 1.011)	1.013 (1.009, 1.017)	1.010 (1.003, 1.018)	1.009 (1.003, 1.016)	1.006 (1.001, 1.011)	1.011 (1.006, 1.015)
CO	1.018 (1.010, 1.026)	1.031 (1.019, 1.043)	1.049 (1.026, 1.073)	1.008 (0.988, 1.029)	1.034 (1.019, 1.050)	1.002 (0.988, 1.016)

^a^Single-pollutant model: adjusted for temperature and humidity;

^b^Two-pollutant models: Model a additionally adjusted for gaseous pollutants (NO_2_, SO_2_ or CO) separately

## Discussion

To the best of our knowledge, this is the first study in China that has examined the adverse effects of PM_2.5_ on hospital admissions based on an entire population of a megalopolis using LUR models. Evidence gained in this study showed a significant PM_2.5_-associated risk on cardiovascular diseases (including hypertension, CHD, and stroke) and respiratory diseases (including COPD), with robust outcomes after adjustment for other gaseous pollutants. Regarding subgroup analyses, the relationship estimates of different age groups varied from each other, while risk estimates were higher in the cold season. These findings provide strong evidence of associations between ambient PM_2.5_ and cardiorespiratory hospital admissions in Wuhan, and might help public agencies to develop strategies for air pollution control and disease prevention.

Our study found that short-term exposure to PM_2.5_ was positively correlated with hospital admissions for respiratory illnesses. However, as PM_2.5_ concentrations in Wuhan were much higher, the magnitude of the risk estimates in our study was generally lower than in prior reports [24, 25]. One multicity study found a strong PM_2.5_-related admission risk at lag2–5 in five European cities, in which a 12.4 μg/m^3^ increase in PM_2.5_ was associated with a 6.4% increase in respiratory hospital admissions [25]. Several explanations might account for the spatial heterogeneity of air pollution-associated health effects. First, as shown in the previous study, the exposure–response relationship between health outcomes and fine particulate matter is relatively steep at low levels of exposure and flattens out at higher exposures [26]. Individuals vulnerable to air pollution might have developed symptoms and gone to the hospital before air pollution concentrations reached a high level [27]. Second, compared with the developed countries, Wuhan has a younger age structure, making it less sensitive to exposure to air pollutants. Moreover, different climate conditions, PM_2.5_ compositions and different lifestyle patterns are possible explanations.

The lag effects of short-term exposure to air pollution are of wide interest in air pollution epidemiology. In this study, PM_2.5_ exhibited a similar lagged pattern for overall cardiorespiratory admission as well as in different subgroups. For single-day lag models, the estimates for PM_2.5_ were the highest at lag0 day and decreased in later lag days, in line with previous studies [10, 28]. This temporal pattern suggests that exposure to PM_2.5_ may increase the risk of hospital admission within hours of exposure. A multi-city study in England and Wales found an elevated risk for myocardial infarction by PM_10_ and NO_2_ at lag1–6 h, with excess risks of 1.2% (95% CI 0.3–2.1%) and 1.1% (95% CI 0.3–2.1%) respectively, per 10 μg/m^3^ increase [29]. Another study from Japan also found that hourly changes in particulate matter (0 to < 6 h) were positively associated with the risk of cardiovascular and cerebrovascular disease [30]. In the present investigation, we found that a moving average lag model usually had higher estimates than that of single-day exposure, with the greatest effects observed at lag0–2. Similar results have also been observed in other continents [8, 31]. In New England, a study found that the highest harmful effects of PM_2.5_ exposure were at lag0–5, for each 10 μg/m^3^ increment, associated with an increase of 4.31, 3.95, and 2.56% percentage change in the hospital admission rates for myocardial infarction, congestive heart failure, and ischemic stroke respectively [8]. Another study in Denmark found the highest ultrafine particle associated risk for stroke at lag0–4 [31]. The variation in days of moving average pattern could be due to the combined effects of many complex factors such as different types of disease, individual behavioral patterns, air pollution components, and the differences in study design. These findings suggest that the effects of air pollution across several days impact daily hospital admissions. There is also experimental support for this pattern, as a toxicological study reported that acute lung inflammation, induced by particle instillation, took up to 4 days to resolve [32]. Considering that the time scale extends over several days, a moving average lag model might be a better exposure metric than a single-day lag in air pollution epidemiological studies. These results provide solid evidence about the importance of the timing of air pollution exposure.

This study explored the demographic-specific associations between PM_2.5_ and hospital admissions for respiratory and cardiovascular diseases. Similar to other studies, a higher susceptibility to PM_2.5_ was found among the elderly (over 65 years old). Such elderly high-risk association is widely accepted due to the weaker immune systems and potential for more chronic medical conditions. In addition, interesting deviations from this pattern were observed for specific subgroups of disease. The risk of COPD, coronary heart disease, and stroke in this study peaked in the middle-age group. For cause-specific cardiovascular diseases, CHD and stroke, the adverse effects were slightly higher in the 65–74 years group than in the over-74 years group. This result may be the consequence of a “harvesting effect” in which susceptible residents might have developed symptoms and died before reaching the age of 75 [33]. Notably, for the COPD patients, stronger associations were found in those aged 45–54 years old, with each 10 μg/m^3^ increase in PM_2.5_ corresponding to a 4.25% (95% CI 1.02%–7.58%) increase in hospital admissions. This finding seems inconsistent to prior study results. In a cohort study conducted in the United States, a higher risk for hospital admission for COPD was found in age group ≥ 76 [34]. More recently, a review of 30 epidemiological studies on air pollution and the morbidity of COPD and asthma found no evidence for the effect of any pollutants on hospital utilization in people aged 15–64 [35]. The variability in these results could be due to possible differences in selection strategy of the study population. As COPD is largely encountered in the elderly, previous studies tended to select people ≥ 65 years old as the study population, or divided age into categorical variables based on 15 and 65 years old [34, 36], which limited the power to examine the relationship between COPD and air pollutants in specific age groups. Further investigations are still needed to explore vulnerable populations.

The assessment of gender differences has been of wide interest in air pollution epidemiology. In the current study, although statistical significance of in gender difference was not observed for PM-associated risks for hospital admission, there were still slight deviations in the magnitude of risk estimates in males and females. For total respiratory disease, slightly higher risk estimates were found in females. For specific cardiovascular diseases, coronary heart disease and stroke, we found that males were at slightly higher risk for hospital admissions. Consistent with the results of the present study, a pooled analysis from 33 Chinese communities reported that the effect of ambient air pollution exposure on the prevalence of stroke and CVD was much higher in men than in women [37]. Another multi-country study in the United States suggested that women might be more susceptible to PM_2.5_-related hospitalizations for respiratory causes [24]. However, these differences could be related to factors such as chemical components and exposure patterns of local populations. The findings of the current study indicated that gender difference tendencies for PM-associated risk may vary among different diseases. The underlying pathology and mechanism of these discrepancies should be further explored in future investigations so as to protect vulnerable subpopulations from PM pollution.

In this study, higher short-term effects of PM_2.5_ on cardiorespiratory hospital admissions were found during the cold period. This may be due to the seasonal variation of PM_2.5_ in Wuhan, with a high concentration in winter and a lower one in summer (Fig. 2), combined with a sharper response at higher concentrations in the exposure response curve (Fig. 5). Relatively low temperatures in the winter can accelerate the conversion of particles, while low wind speed restricts air pollutants from dispersing [38]. The seasonal finding in this study echoes a study in Hong Kong [39], which found an increased risk of respiratory mortality in the cold season when PM_10_ concentrations were up to 80 μg/m^3^. Two previous large-scale analyses from the United States also found larger PM_2.5_-induced risks of hospitalizations for cardiovascular and respiratory diseases in cold months (winter or spring) [24, 40].

In two-pollutant analyses, the associations of PM_2.5_ with total cardiovascular and respiratory diseases remained robust after adjustment for gaseous pollutants (NO_2_, SO_2_ and CO), suggesting PM_2.5_ seemingly has independent impact on total cardiorespiratory hospital admission. We also observed that the effect estimates of PM_2.5_ in two-pollutant model were slightly smaller than in single-pollutant model. In addition, after adjusting for PM_2.5_, gaseous pollutants themselves have an adverse impact on most cardiorespiratory diseases. Many studies have indicated similar results with this one [41–43]. Possible reason could be the confounding effect of gaseous pollutants. The observed effects of PM_2.5_ using single-pollutant models might be partly due to the exposure of gaseous pollutants, while the independent effect of gaseous pollutants has been proved [44]. However, these findings should be interpreted with caution, because the high correlation amongst the pollutants may render the model partly unstable.

Compared with previous studies, this study has several strengths. First, we obtained hospitalization data from a total of 59 hospitals in Wuhan to evaluate the PM-admission relationships. Given Wuhan’s universal access to hospital health care, the potential for selection bias was minimized and the results can be directly generalized to the whole city. Second, the adoption of LUR model increases the accuracy when assessing the spatial variations in individual PM_2.5_ exposures and in detecting possible associations. This study has some limitations as well. First, we linked PM_2.5_ to cardiorespiratory diseases by date of hospital admission rather than by the date of symptom onset. This may have introduced a non-differential error in exposure measurement and underestimated the effect estimates. Second, while the exposure modeling methods employed in this study added new information in comparison with most previous studies, the deficiency of PM_2.5_ exposure data from occupation, commuting, and pollution originating from indoor sources may have further attenuated our effect estimates. Third, although the two-pollutant models were fitted to examine the robustness of the association between PM_2.5_ and hospital admissions, the collinearity between pollutants limited the ability to separate the independent effect of PM_2.5_.

## Conclusion

This study provides evidence regarding the short-term health impacts of PM_2.5_ exposure as well as identifies sensitive subpopulations in Wuhan. We find that the cumulative effect of short-term PM_2.5_ exposure are higher than that of single day. The risk estimates of different age groups vary from each other, while the elderly are still at higher risk for most diseases. Besides, the higher PM-induced risk during the cold season cannot be ignored. These findings extend our knowledge related to the effects of higher levels of exposure and may help public agencies to develop strategies for air pollution control.

## Supplementary Information

**Additional file 1:** **Figure S1.** Flow chart of the selection process for the study population.

**Additional file 2:** **Table S1.** Description of Developed LUR Models for PM2.5 in different year.

**Additional file 3:** **Table S2.** Descriptive statistics of air pollutant concentration and meteorological factor in Wuhan, 2016.10 -2018.12.

**Additional file 4:** **Table S3.** Spearman correlations among environmental variables in Wuhan, 2016.10 -2018.12.

**Additional file 5:** **Table S4.** Odds ratio (95% CIs) of admissions at various exposure days, associated with per 10 μg/m^3^ increase of PM_2.5_.

**Additional file 6:** **Table S5.** Odds ratio (95% CIs) of admissions at various exposure days, associated with per 10 μg/m^3^ increase of PM_2.5_, using symmetric CCO design.

**Additional file 7:** **Table S6.** Sensitive analyses of odds ratio (95% CIs) of admissions at lag0~2 under varying degrees of freedom (df) for, associated with per 10 μg/m^3^ increase of PM_2.5_.

**Additional file 8:** **Table S7.** Odds ratio (95% CIs) of cardiorespiratory hospital admissions stratified by age, gender and season, associated with per 10 μg/m3 increase of PM_2.5_.

## Data Availability

The data that support the findings of this study are available from the Wuhan Information Center of Health and Family Planning, but restrictions apply to the availability of these data, which were used under license for the current study, and so are not publicly available. Data are however available from the authors upon reasonable request and with permission of the Wuhan Information Center of Health and Family Planning.

## Abbreviations

AQG: Air quality guidelines
AQS: Ambient air quality standards
CCO: Case-crossover
CHD: Coronary heart disease
CI: Confidence interval
CO: Carbon monoxide
COPD: Chronic obstructive pulmonary disease
CTM: Atmospheric chemical transport model
CVD: Cardiovascular disease
DALYs: Disability-adjusted life-years
DOW: Day of the week
DF: Degrees of freedom
DM: Dispersion models
GIS: Geographic information system
LUR: Land use regression
NCS: Natural cubic spline
NO_2_: Nitrogen dioxide
OR: Odds ratio
PM_10_: Particulate matter with an aerodynamic diameter of 10 μm or less
PM_2.5_: Particulate matter with an aerodynamic diameter of 2.5 μm or less
SD: Standard deviations
SO_2_: Sulfur dioxide
WHO: World Health Organization
