# Supplementary figures and images for: Effect of ambient fine particulates (PM2.5) on hospital admissions for respiratory and cardiovascular diseases in Wuhan, China
Source: Respir Res. 2021 Apr 28;22:128. doi: 10.1186/s12931-021-01731-x (PMC8080330; doi:10.1186/s12931-021-01731-x)

**Additional file**


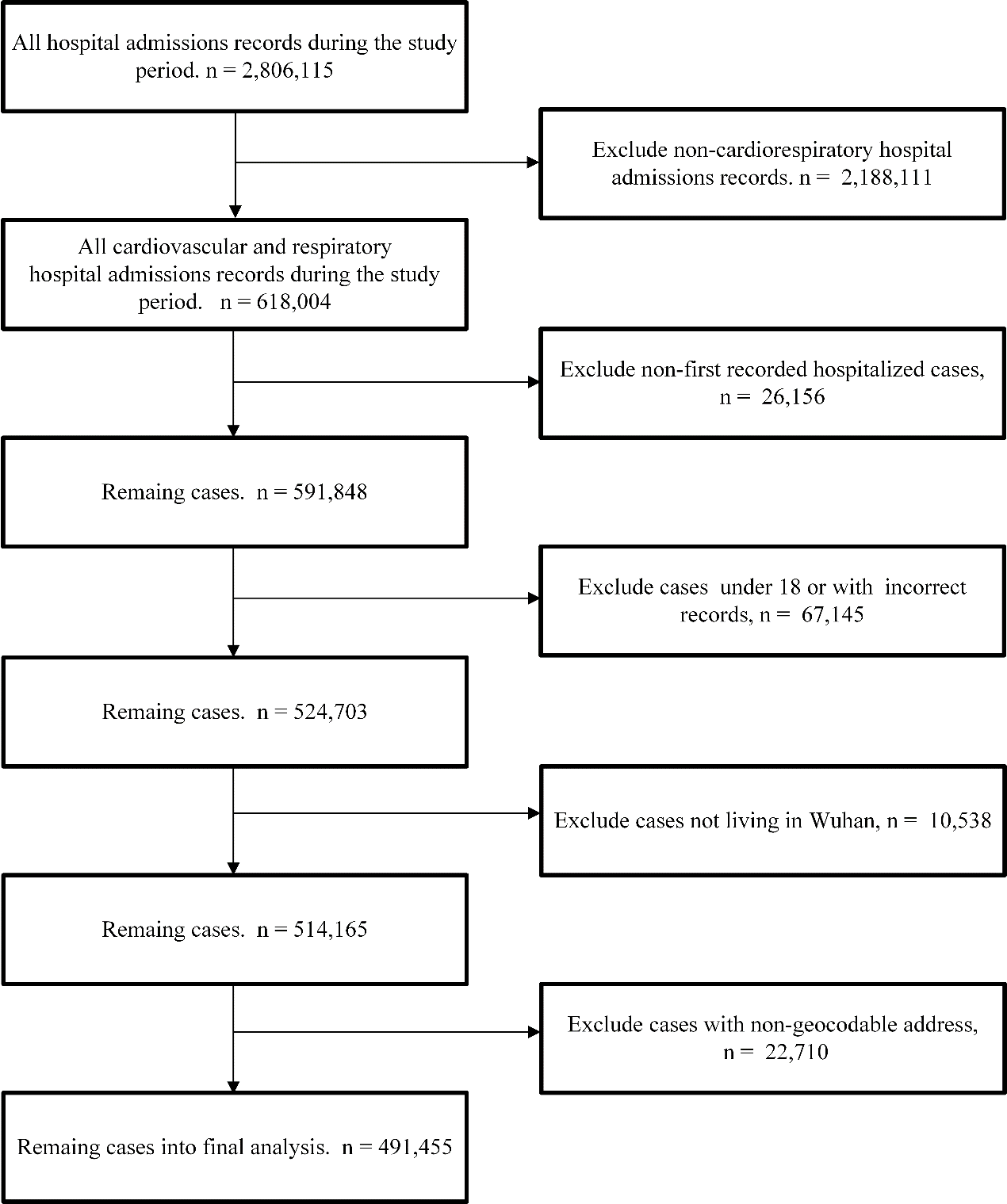


**Figure S1**. Flow chart of the selection process for the study population

Supplement: Supplementary file 1 — Additional file 1: Figure S1. Flow chart of the selection process for the study population. [file 12931_2021_1731_MOESM1_ESM.docx]
