# Supplementary material for: Effect of ambient fine particulates (PM2.5) on hospital admissions for respiratory and cardiovascular diseases in Wuhan, China
Source: Respir Res. 2021 Apr 28;22:128. doi: 10.1186/s12931-021-01731-x (PMC8080330; doi:10.1186/s12931-021-01731-x)
Supplement: Supplementary file 2 — Additional file 2: Table S1. Description of Developed LUR Models for PM2.5 in different year. [file 12931_2021_1731_MOESM2_ESM.docx]

**Additional file**

We developed the LUR model using a range of geographic predictors, including types of land use (<https://earthexplorer.usgs.gov/>), the length of roads (<http://www.openstreetmap.org>), the nearest distance between the station and the road, the number of industrial sources (<http://www.whepb.gov.cn/>), population density (<https://sedac.ciesin.columbia.edu/>), and digital elevation (<http://srtm.csi.cgiar.org>). Considering the long span of our research period, we use the annual average concentration for modeling to better assess the individual’s exposure. According to the standardized procedure of the European Study of Cohorts for Air Pollution Effects (ESCAPE), the LUR model was developed based on the one-year annual mean PM_2.5_ concentrations of 16 monitoring sites. Therefore, three LUR models were built respectively, each of which explained at least 80% of the variability of the monitor concentration at a fixed site, and yielded high leave-one-out-cross-validation (LOOCV) R^2^ value (Table S1).

| **Table S1**. Description of Developed LUR Models for PM_2.5_ in different year. | | | | |
| --- | --- | --- | --- | --- |
| Study period | LUR model ^a^ | R^2^ of model | R^2^ validation | RMSE (validation) (μg/m^3^) |
| 2016.10.1~2016.12.31 | 47.72 + 1.99 × INDUNUM_7000 + 0.81×10^-2^ × SECOND_1000 + 91285.35 × MAJORDISTINVNEARC2 | 0.84 | 0.61 | 4.02 |
| 2017.1.1~2017.12.31 | 43.44 + 5.57 × 10^-6^ × RL_500 + 7.63×10^-5^ × SECOND_3000 + 99298.59 × MAJORDISTINVNEARC2 | 0.86 | 0.67 | 2.32 |
| 2018.1.1~2018.12.31 | 34.69 + 2.95 × INDUNUM_7000 + 0.44×10^-3^ × SECOND_1000 + 16775.63 × TERTIARYDISTINVNEARC2 | 0.81 | 0.73 | 2.62 |
| ^a^ Some variables are buffers with _X indicating the radius of the buffer in meters Abbreviation: INDUNUM: industry number; RL: residential land; SECOND: Secondary highway; MAJORDISTINVNEARC2: inverse squared distance to the nearest major road; TERTIARYDISTINVNEARC2: inverse squared distance to the tertiary highway. | | | | |
