# Supplementary material for: Effect of ambient fine particulates (PM2.5) on hospital admissions for respiratory and cardiovascular diseases in Wuhan, China
Source: Respir Res. 2021 Apr 28;22:128. doi: 10.1186/s12931-021-01731-x (PMC8080330; doi:10.1186/s12931-021-01731-x)
Supplement: Supplementary file 3 — Additional file 3: Table S2. Descriptive statistics of air pollutant concentration and meteorological factor in Wuhan, 2016.10 -2018.12. [file 12931_2021_1731_MOESM3_ESM.docx]

**Additional file**

| **Table S2**. Descriptive statistics of air pollutant concentration and meteorological factor in Wuhan, 2016.10 -2018.12. | | | | | | | |
| --- | --- | --- | --- | --- | --- | --- | --- |
| Variable | Mean ± SD | Min | Percentile | | | Max |  |
|  |  |  | P25 | P50 | P75 |  |  |
| Air pollutant |  |  |  |  |  |  |  |
| PM_2.5_ (μg/m^3^) | 48.2 ± 30.6 | 2.5 | 26.6 | 41.4 | 62.2 | 238.0 |  |
| SO_2_ (μg/m^3^) | 28.5 ± 17.6 | 3.0 | 15.0 | 24.0 | 36.0 | 135.0 |  |
| NO_2_ (μg/m^3^) | 43.3 ± 23.3 | 2.4 | 26.4 | 39.2 | 57.6 | 154.0 |  |
| CO (μg/m^3^) | 2.4 ± 1.0 | 0.5 | 1.8 | 2.3 | 3.0 | 9.4 |  |
| Meteorological factor |  |  |  |  |  |  |  |
| Mean temperature (°C) | 16.3 ± 9.4 | -3.8 | 8.8 | 16.7 | 24.4 | 33.9 |  |
| Relative humidity (%) | 80 ± 10 | 25 | 73 | 81 | 88 | 100 |  |
