# Supplementary material for: Effect of ambient fine particulates (PM2.5) on hospital admissions for respiratory and cardiovascular diseases in Wuhan, China
Source: Respir Res. 2021 Apr 28;22:128. doi: 10.1186/s12931-021-01731-x (PMC8080330; doi:10.1186/s12931-021-01731-x)
Supplement: Supplementary file 4 — Additional file 4: Table S3. Spearman correlations among environmental variables in Wuhan, 2016.10 -2018.12. [file 12931_2021_1731_MOESM4_ESM.docx]

**Additional file**

| **Table S3**. Spearman correlations among environmental variables in Wuhan, 2016.10 -2018.12. | | | | | | |
| --- | --- | --- | --- | --- | --- | --- |
| Variable | PM_2.5_ | SO_2_ | NO_2_ | CO | tem | rh |
| PM_2.5_ | 1 |  |  |  |  |  |
| SO_2_ | 0.67** | 1 |  |  |  |  |
| NO_2_ | 0.70** | 0.79** | 1 |  |  |  |
| CO | 0.76** | 0.54** | 0.62** | 1 |  |  |
| tem | -0.55** | -0.45** | -0.39** | -0.4 | 1 |  |
| rh | -0.13 | -0.44** | -0.19** | 0.15** | -0.07 | 1 |
| ** Statistical significantly (p < 0.01). | | | | | | |
