# Supplementary material for: Effect of ambient fine particulates (PM2.5) on hospital admissions for respiratory and cardiovascular diseases in Wuhan, China
Source: Respir Res. 2021 Apr 28;22:128. doi: 10.1186/s12931-021-01731-x (PMC8080330; doi:10.1186/s12931-021-01731-x)
Supplement: Supplementary file 5 — Additional file 5: Table S4. Odds ratio (95% CIs) of admissions at various exposure days, associated with per 10 μg/m3 increase of PM2.5. [file 12931_2021_1731_MOESM5_ESM.docx]

| **Table S4**. Odds ratio (95% CIs) of admissions at various exposure days, associated with per 10 μg/m^3^ increase of PM_2.5_ | | | | | | | | | | | | | | | | | |  |
| --- | --- | --- | --- | --- | --- | --- | --- | --- | --- | --- | --- | --- | --- | --- | --- | --- | --- | --- |
| Lagdays | CVD | |  | Respiratory | |  | COPD | |  | Hypertension | |  | CHD | |  | Stroke | | |
|  | OR | 95%CI |  | OR | 95%CI |  | OR | 95%CI |  | OR | 95%CI |  | OR | 95%CI |  | OR | 95%CI | |
| lag0 | 1.0084 | (1.0067, 1.0101) |  | 1.0140 | (1.0116, 1.0164) |  | 1.0138 | (1.0092, 1.0184) |  | 1.0078 | (1.0037, 1.0119) |  | 1.0085 | (1.0054, 1.0116) |  | 1.0078 | (1.0078, 1.0048) | |
| lag1 | 1.0082 | (1.0065, 1.0099) |  | 1.0118 | (1.0093, 1.0142) |  | 1.0112 | (1.0066, 1.0158) |  | 1.0095 | (1.0053, 1.0137) |  | 1.0080 | (1.0049, 1.0112) |  | 1.0073 | (1.0073, 1.0043) | |
| lag2 | 1.0043 | (1.0026, 1.0060) |  | 1.0081 | (1.0057, 1.0105) |  | 1.0094 | (1.0049, 1.0140) |  | 1.0051 | (1.0010, 1.0093) |  | 1.0063 | (1.0031, 1.0094) |  | 1.0031 | (1.0031, 1.0001) | |
| lag3 | 1.0025 | (1.0009, 1.0042) |  | 1.0037 | (1.0013, 1.0061) |  | 1.0027 | (0.9982, 1.0072) |  | 1.0021 | (0.9980, 1.0062) |  | 1.0037 | (1.0007, 1.0068) |  | 1.0026 | (1.0026, 0.9996) | |
| lag4 | 1.0012 | (0.9995, 1.0029) |  | 1.0025 | (1.0001, 1.0049) |  | 1.0026 | (0.9980, 1.0072) |  | 1.0036 | (0.9994, 1.0078) |  | 1.0013 | (0.9981, 1.0044) |  | 1.0002 | (1.0002, 0.9972) | |
| lag5 | 0.9999 | (0.9981, 1.0017) |  | 1.0048 | (1.0023, 1.0073) |  | 1.0036 | (0.9988, 1.0083) |  | 1.0026 | (0.9982, 1.0069) |  | 0.9980 | (0.9948, 1.0012) |  | 1.0007 | (1.0007, 0.9976) | |
| lag6 | 0.9986 | (0.9968, 1.0004) |  | 1.0030 | (1.0004, 1.0055) |  | 1.0040 | (0.9992, 1.0088) |  | 1.0022 | (0.9978, 1.0066) |  | 0.9974 | (0.9941, 1.0007) |  | 0.9966 | (0.9966, 0.9935) | |
| lag0~1 | 1.0116 | (1.0096, 1.0136) |  | 1.0174 | (1.0146, 1.0202) |  | 1.0171 | (1.0117, 1.0224) |  | 1.0111 | (1.0063, 1.0159) |  | 1.0115 | (1.0079, 1.0151) |  | 1.0107 | (1.0107, 1.0073) | |
| lag0~2 | 1.0123 | (1.0101, 1.0145) |  | 1.0195 | (1.0163, 1.0226) |  | 1.0202 | (1.0143, 1.0262) |  | 1.0112 | (1.0059, 1.0166) |  | 1.0132 | (1.0092, 1.0172) |  | 1.0110 | (1.0110, 1.0072) | |
| lag0~3 | 1.0116 | (1.0092, 1.014) |  | 1.0188 | (1.0154, 1.0222) |  | 1.0186 | (1.0122, 1.0251) |  | 1.0093 | (1.0036, 1.0151) |  | 1.0132 | (1.0088, 1.0175) |  | 1.0106 | (1.0106, 1.0064) | |
| lag0~4 | 1.0107 | (1.0081, 1.0132) |  | 1.0183 | (1.0146, 1.0219) |  | 1.0180 | (1.0111, 1.0250) |  | 1.0088 | (1.0026, 1.0150) |  | 1.0123 | (1.0076, 1.0170) |  | 1.0093 | (1.0093, 1.0048) | |
| lag0~5 | 1.0096 | (1.0069, 1.0124) |  | 1.0190 | (1.0151, 1.0229) |  | 1.0178 | (1.0103, 1.0253) |  | 1.0081 | (1.0015, 1.0148) |  | 1.0102 | (1.0052, 1.0153) |  | 1.0087 | (1.0087, 1.0039) | |
| lag0~6 | 1.0084 | (1.0055, 1.0114) |  | 1.0196 | (1.0153, 1.0239) |  | 1.0186 | (1.0104, 1.0268) |  | 1.0078 | (1.0006, 1.0151) |  | 1.0083 | (1.0028, 1.0138) |  | 1.0071 | (1.0071, 1.0018) | |

**Additional file**
