# Supplementary material for: Effect of ambient fine particulates (PM2.5) on hospital admissions for respiratory and cardiovascular diseases in Wuhan, China
Source: Respir Res. 2021 Apr 28;22:128. doi: 10.1186/s12931-021-01731-x (PMC8080330; doi:10.1186/s12931-021-01731-x)
Supplement: Supplementary file 6 — Additional file 6: Table S5. Odds ratio (95% CIs) of admissions at various exposure days, associated with per 10 μg/m3 increase of PM2.5, using symmetric CCO design. [file 12931_2021_1731_MOESM6_ESM.docx]

**Additional file**

| **Table S5**. Odds ratio (95% CIs) of admissions at various exposure days, associated with per 10 μg/m^3^ increase of PM_2.5_, using symmetric CCO design. | | | | | | | | | | | | | | | | | |
| --- | --- | --- | --- | --- | --- | --- | --- | --- | --- | --- | --- | --- | --- | --- | --- | --- | --- |
| lagdays | CVD | |  | Respiratory | |  | COPD | |  | Hypertension | |  | CHD | |  | Stroke | |
|  | OR | 95%CI |  | OR | 95%CI |  | OR | 95%CI |  | OR | 95%CI |  | OR | 95%CI |  | OR | 95%CI |
| lag0 | 1.007 | (1.005, 1.008) |  | 1.013 | (1.011, 1.016) |  | 1.014 | (1.009, 1.018) |  | 1.007 | (1.003, 1.011) |  | 1.006 | (1.003, 1.009) |  | 1.007 | (1.004, 1.009) |
| lag1 | 1.005 | (1.004, 1.007) |  | 1.011 | (1.009, 1.013) |  | 1.011 | (1.007, 1.016) |  | 1.005 | (1.001, 1.009) |  | 1.004 | (1.001, 1.007) |  | 1.006 | (1.003, 1.009) |
| lag2 | 1.003 | (1.001, 1.004) |  | 1.009 | (1.007, 1.011) |  | 1.010 | (1.005, 1.014) |  | 1.003 | (0.999, 1.007) |  | 1.003 | (1.001, 1.006) |  | 1.003 | (0.999, 1.006) |
| lag3 | 1.002 | (1.001, 1.004) |  | 1.006 | (1.003, 1.008) |  | 1.006 | (1.001, 1.010) |  | 1.001 | (0.996, 1.004) |  | 1.002 | (0.999, 1.005) |  | 1.004 | (1.001, 1.007) |
| lag4 | 1.003 | (1.001, 1.005) |  | 1.005 | (1.003, 1.007) |  | 1.006 | (1.001, 1.010) |  | 1.003 | (0.999, 1.007) |  | 1.002 | (0.999, 1.005) |  | 1.004 | (1.001, 1.007) |
| lag5 | 0.997 | (0.995, 0.999) |  | 1.002 | (0.999, 1.005) |  | 1.001 | (0.996, 1.005) |  | 0.998 | (0.994, 1.002) |  | 0.995 | (0.992, 0.998) |  | 0.998 | (0.995, 1.001) |
| lag6 | 0.992 | (0.991, 0.994) |  | 0.996 | (0.994, 0.999) |  | 0.997 | (0.992, 1.001) |  | 0.994 | (0.990, 0.999) |  | 0.991 | (0.987, 0.994) |  | 0.992 | (0.989, 0.995) |
| lag0~1 | 1.009 | (1.007, 1.011) |  | 1.016 | (1.014, 1.019) |  | 1.017 | (1.012, 1.022) |  | 1.008 | (1.003, 1.012) |  | 1.007 | (1.004, 1.011) |  | 1.009 | (1.006, 1.012) |
| lag0~2 | 1.009 | (1.007, 1.011) |  | 1.019 | (1.016, 1.022) |  | 1.020 | (1.014, 1.025) |  | 1.008 | (1.002, 1.013) |  | 1.008 | (1.004, 1.012) |  | 1.009 | (1.006, 1.013) |
| lag0~3 | 1.008 | (1.006, 1.010) |  | 1.019 | (1.016, 1.022) |  | 1.019 | (1.013, 1.025) |  | 1.005 | (0.999, 1.011) |  | 1.008 | (1.004, 1.012) |  | 1.009 | (1.005, 1.013) |
| lag0~4 | 1.008 | (1.006, 1.011) |  | 1.019 | (1.016, 1.023) |  | 1.019 | (1.013, 1.026) |  | 1.005 | (0.999, 1.011) |  | 1.008 | (1.003, 1.012) |  | 1.001 | (1.005, 1.014) |
| lag0~5 | 1.006 | (1.004, 1.009) |  | 1.019 | (1.015, 1.023) |  | 1.018 | (1.011, 1.026) |  | 1.003 | (0.997, 1.010) |  | 1.005 | (1.001, 1.010) |  | 1.008 | (1.004, 1.013) |
| lag0~6 | 1.003 | (1.001, 1.006) |  | 1.018 | (1.014, 1.021) |  | 1.017 | (1.009, 1.024) |  | 1.001 | (0.994, 1.007) |  | 1.001 | (0.996, 1.006) |  | 1.005 | (1.001, 1.010) |
