# Supplementary material for: Effect of ambient fine particulates (PM2.5) on hospital admissions for respiratory and cardiovascular diseases in Wuhan, China
Source: Respir Res. 2021 Apr 28;22:128. doi: 10.1186/s12931-021-01731-x (PMC8080330; doi:10.1186/s12931-021-01731-x)
Supplement: Supplementary file 7 — Additional file 7: Table S6. Sensitive analyses of odds ratio (95% CIs) of admissions at lag0~2 under varying degrees of freedom (df) for, associated with per 10 μg/m3 increase of PM2.5. [file 12931_2021_1731_MOESM7_ESM.docx]

**Additional file**

| **Table S6**. Sensitive analyses of odds ratio (95% CIs) of admissions at lag0~2 under varying degrees of freedom (df) for, associated with per 10 μg/m^3^ increase of PM_2.5_. | | | | | | | | | | | | |
| --- | --- | --- | --- | --- | --- | --- | --- | --- | --- | --- | --- | --- |
| Temperature df | Humidity df | CVD |  | Respiratory |  | COPD |  | Hypertension |  | CHD |  | Stroke |
| 2 | 2 | 1.014 (1.012, 1.016) |  | 1.020 (1.017, 1.023) |  | 1.020 (1.014, 1.026) |  | 1.014 (1.009, 1.020) |  | 1.015 (1.011, 1.019) |  | 1.012 (1.008, 1.016) |
| 2 | 3 | 1.013 (1.011, 1.015) |  | 1.019 (1.016, 1.023) |  | 1.020 (1.014, 1.026) |  | 1.013 (1.008, 1.018) |  | 1.014 (1.010, 1.018) |  | 1.011 (1.008, 1.015) |
| 2 | 4 | 1.013 (1.011, 1.015) |  | 1.019 (1.016, 1.022) |  | 1.019 (1.014, 1.025) |  | 1.013 (1.007, 1.018) |  | 1.014 (1.010, 1.018) |  | 1.011 (1.007, 1.015) |
| 3 | 2 | 1.012 (1.010, 1.015) |  | 1.019 (1.016, 1.022) |  | 1.020 (1.014, 1.026) |  | 1.012 (1.007, 1.017) |  | 1.013 (1.009, 1.017) |  | 1.011 (1.007, 1.015) |
| 3 | 4 | 1.011 (1.009, 1.014) |  | 1.019 (1.016, 1.022) |  | 1.019 (1.013, 1.025) |  | 1.010 (1.005, 1.016) |  | 1.013 (1.009, 1.017) |  | 1.010 (1.006, 1.014) |
| 4 | 2 | 1.013 (1.010, 1.015) |  | 1.019 (1.016, 1.022) |  | 1.020 (1.014, 1.026) |  | 1.013 (1.007, 1.018) |  | 1.014 (1.010, 1.018) |  | 1.011 (1.007, 1.015) |
| 4 | 3 | 1.012 (1.010, 1.014) |  | 1.019 (1.016, 1.022) |  | 1.020(1.014, 1.026) |  | 1.011 (1.006, 1.017) |  | 1.013 (1.009, 1.017) |  | 1.011 (1.007, 1.015) |
| 4 | 4 | 1.012 (1.009, 1.014) |  | 1.019 (1.016, 1.022) |  | 1.019 (1.013, 1.025) |  | 1.011 (1.006, 1.016) |  | 1.013 (1.009, 1.017) |  | 1.01 (1.006, 1.014) |
