# Supplementary material for: Effect of ambient fine particulates (PM2.5) on hospital admissions for respiratory and cardiovascular diseases in Wuhan, China
Source: Respir Res. 2021 Apr 28;22:128. doi: 10.1186/s12931-021-01731-x (PMC8080330; doi:10.1186/s12931-021-01731-x)
Supplement: Supplementary file 8 — Additional file 8: Table S7. Odds ratio (95% CIs) of cardiorespiratory hospital admissions stratified by age, gender and season, associated with per 10 μg/m3 increase of PM2.5. [file 12931_2021_1731_MOESM8_ESM.docx]

**Additional file**

| **Table S7**. Odds ratio (95% CIs) of cardiorespiratory hospital admissions stratified by age, gender and season , associated with per 10 μg/m^3^ increase of PM_2.5_ | | | | | | | | | | | | | | | | | |
| --- | --- | --- | --- | --- | --- | --- | --- | --- | --- | --- | --- | --- | --- | --- | --- | --- | --- |
| Subgroup | CVD | |  | Respiratory | |  | COPD | |  | Hypertension | |  | CHD | |  | Stroke | |
|  | OR (95%CI) | P value |  | OR (95%CI) | P value |  | OR (95%CI) | P value |  | OR (95%CI) | P value |  | OR (95%CI) | P value |  | OR (95%CI) | P value |
| Age group | |  |  |  |  |  |  |  |  |  |  |  |  |  |  |  |  |
| <45 | 1.003 (0.994, 1.012) | - |  | 1.010 (1.002, 1.019) | - |  | 0.989 (0.953, 1.028) | - |  | 1.010 (0.991, 1.030) | - |  | 1.003 (0.978, 1.028) | - |  | 1.004 (0.984, 1.025) | - |
| 45~54 | 1.008 (1.002, 1.014) | 0.3639 |  | 1.021 (1.011, 1.031) | 0.0971 |  | 1.042 (1.010, 1.075) | 0.0373 |  | 1.001 (0.986, 1.013) | 0.3893 |  | 1.011 (0.999, 1.023) | 0.5675 |  | 1.006 (0.995, 1.018) | 0.7251 |
| 55~64 | 1.011 (1.006, 1.015) | 0.1153 |  | 1.018 (1.011, 1.025) | 0.1545 |  | 1.019 (1.004, 1.034) | 0.1504 |  | 1.009 (0.998, 1.020) | 0.9101 |  | 1.014 (1.006, 1.022) | 0.4089 |  | 1.008 (1.001, 1.016) | 0.2904 |
| 65~74 | 1.014 (1.010, 1.018) | 0.0289 |  | 1.019 (1.013, 1.026) | 0.0916 |  | 1.022 (1.011, 1.033) | 0.0975 |  | 1.012 (1.000, 1.023) | 0.8935 |  | 1.013 (1.006, 1.021) | 0.4387 |  | 1.014 (1.007, 1.022) | 0.0081 |
| >74 | 1.014 (1.010, 1.018) | 0.0263 |  | 1.022 (1.017, 1.028) | 0.0162 |  | 1.018 (1.010, 1.026) | 0.1411 |  | 1.017 (1.008, 1.027) | 0.5024 |  | 1.012 (1.005, 1.019) | 0.4969 |  | 1.011 (1.005, 1.018) | 0.0292 |
| Gender | |  |  |  |  |  |  |  |  |  |  |  |  |  |  |  |  |
| Men | 1.011 (1.008, 1.014) | - |  | 1.017 (1.013, 1.021) | - |  | 1.019 (1.012, 1.026) | - |  | 1.007 (0.999, 1.014) | - |  | 1.014 (1.008, 1.019) | - |  | 1.012 (1.007, 1.017) | - |
| Women | 1.012 (1.009, 1.015) | 0.7552 |  | 1.022 (1.017, 1.027) | 0.1223 |  | 1.023 (1.011, 1.035) | 0.5838 |  | 1.015 (1.008, 1.023) | 0.1156 |  | 1.012 (1.006, 1.018) | 0.6003 |  | 1.009 (1.003, 1.015) | 0.3950 |
| Season at admission | |  |  |  |  |  |  |  |  |  |  |  |  |  |  |  |  |
| Warm | 1.005 (1.000, 1.011) | - |  | 1.014 (1.006, 1.022) | - |  | 1.007 (0.991, 1.022) | - |  | 1.004 (0.990, 1.018) | - |  | 1.004 (0.994, 1.014) | - |  | 1.002 (0.993, 1.011) | - |
| Cold | 1.012 (1.009, 1.014) | 0.0312 |  | 1.020 (1.016, 1.023) | 0.1879 |  | 1.021 (1.015, 1.028) | 0.0877 |  | 1.010 (1.004, 1.016) | 0.4160 |  | 1.013 (1.008, 1.017) | 0.1197 |  | 1.012 (1.008, 1.016) | 0.0546 |
